# Bis[μ-3-(2-hy­droxy­phen­yl)propenoato]bis­{aqua­(4,4′-bipyridine)­bis­[3-(2-hydroxy­phen­yl)propenoato]yttrium(III)} 4,4′-bipyridine disolvate

**DOI:** 10.1107/S1600536810044831

**Published:** 2010-11-06

**Authors:** Chun-Yan Zhang, Jun-Dan Fu, Yi-Hang Wen

**Affiliations:** a Zhejiang Key Laboratory for Reactive Chemistry on Solid Surfaces, Institute of Physical Chemistry, Zhejiang Normal University, Jinhua, Zhejiang 321004, People’s Republic of China

## Abstract

The title compound, [Y_2_(C_9_H_7_O_3_)_6_(C_10_H_8_N_2_)_2_(H_2_O)_2_]·2C_10_H_8_N_2_, contains two eight-coordinated Y^III^ ions, which are linked by two carboxyl­ate groups from two 2-hy­droxy­cinnamate anions, leading to a centrosymmetric dinuclear structure surrounded by solvent 4,4′-bipyridine mol­ecules. It forms a three-dimensional framework connected by extensive O—H⋯O and O—H⋯N hydrogen-bonding inter­actions.

## Related literature

For related compounds, see: Casas *et al.* (2008 ▶); Chowdhury & Kariuki (2006 ▶); Crowther *et al.* (2008 ▶); Darshak *et al.* (2006 ▶); Gossauer *et al.* (2004 ▶).
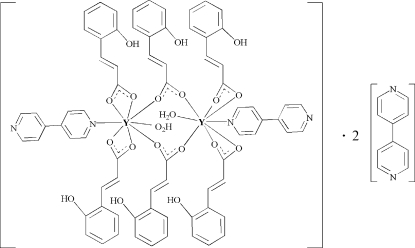


## Experimental

### 

#### Crystal data

[Y_2_(C_9_H_7_O_3_)_6_(C_10_H_8_N_2_)_2_(H_2_O)_2_]·2C_10_H_8_N_2_
*M* *_r_* = 1817.46
Triclinic, 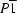

*a* = 11.8464 (7) Å
*b* = 13.5272 (8) Å
*c* = 13.7350 (8) Å
α = 77.561 (3)°
β = 88.850 (3)°
γ = 82.283 (3)°
*V* = 2129.8 (2) Å^3^
*Z* = 1
Mo *K*α radiation
μ = 1.43 mm^−1^
*T* = 296 K
0.18 × 0.16 × 0.13 mm

#### Data collection

Bruker APEXII area-detector diffractometer
Absorption correction: empirical (using intensity measurements) (*SADABS*; Sheldrick, 1996 ▶) *T*_min_ = 0.77, *T*_max_ = 0.83
32270 measured reflections
9803 independent reflections
7446 reflections with *I* > 2σ(*I*)
*R*_int_ = 0.041

#### Refinement

*R*[*F* ^2^ > 2σ(*F* ^2^)] = 0.039
*wR*(*F* ^2^) = 0.087
*S* = 1.02
9803 reflections
574 parameters
6 restraints
H atoms treated by a mixture of independent and constrained refinement
Δρ_max_ = 0.24 e Å^−3^
Δρ_min_ = −0.43 e Å^−3^

### 

Data collection: *APEX2* (Bruker, 2006 ▶); cell refinement: *SAINT* (Bruker, 2006 ▶); data reduction: *SAINT*; program(s) used to solve structure: *SHELXS97* (Sheldrick, 2008 ▶); program(s) used to refine structure: *SHELXL97* (Sheldrick, 2008 ▶); molecular graphics: *SHELXTL* (Sheldrick, 2008 ▶); software used to prepare material for publication: *SHELXTL*.

## Supplementary Material

Crystal structure: contains datablocks I, global. DOI: 10.1107/S1600536810044831/ds2064sup1.cif

Structure factors: contains datablocks I. DOI: 10.1107/S1600536810044831/ds2064Isup2.hkl

Additional supplementary materials:  crystallographic information; 3D view; checkCIF report

## Figures and Tables

**Table 1:** Hydrogen-bond geometry (Å, °)

*D*—H⋯*A*	*D*—H	H⋯*A*	*D*⋯*A*	*D*—H⋯*A*
O1*W*—H1*WA*⋯N4^i^	0.83 (2)	2.00 (2)	2.830 (3)	172 (2)
O9—H9⋯O7^ii^	0.90 (2)	1.77 (2)	2.650 (2)	165 (3)
O1*W*—H1*WB*⋯O5^iii^	0.83 (2)	2.00 (2)	2.814 (2)	166 (3)
O6—H6⋯N2^iv^	0.91 (2)	1.81 (2)	2.708 (3)	167 (3)
O3—H3⋯O4^v^	0.90 (2)	1.72 (2)	2.609 (2)	168 (3)

Symmetry codes: (i) 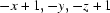; (ii) 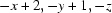; (iii) 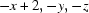; (iv) 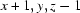; (v) 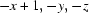.

**Fig. 1.**
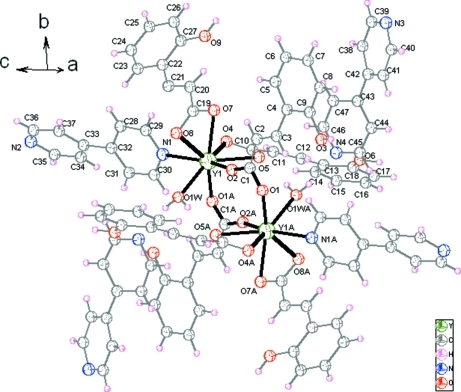
Perspective view of the structure of (I), showing the atom-numbering scheme. Displacement ellipsoids are drawn at the 30% probability level. [Symmetry codes: (a) -x + 2, -y, -z]

**Fig. 2.**
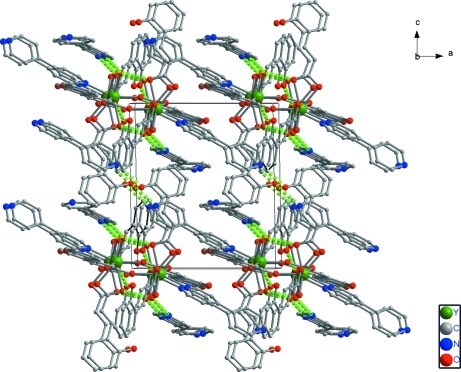
View of the supramolecular network connected by hydrogen bonds along the crystallographic b axis.

## supplementary crystallographic information

### Comment

Very recently, the compounds containing H_2_ca and different metal ions have been reported (Casas *et al.*, 2008; Chowdhury & Kariuki, 2006; Crowther *et al.*, 2008; Darshak *et al.*, 2006; Gossauer *et al.*, 2004). Furthermore, 4,4'-bipy is an excellent bridging ligand in coordination chemistry because of its rod-like shape that allows the ligand to connect metal ions into an extended array. Herein,we report the synthesis and the structure of a new dinuclear yttrium compound [Y_2_(Hca)_6_(4,4'-bipy)_2_(H_2_O)_2_].2(4,4'-bipy), (I), derived from H_2_ca and 4,4'-bipy ligands.

A perspective view of the molecular structure of (I) is presented in Fig. 1. It consists of two Y atoms, six Hca anions, two coordinated water molecules, two coordinated 4,4'-bipy and two lattice included non-coordinated 4,4'-bipy molecules. Two carboxylate group from two Hca anions adopt the bridging mode to bond two Y^III^ ions [Y—O distances: 2.2074 (15) and 2.3143 (15) Å], which lead to the dinuclear structure with Y···Y separation of 4.5721 (4) Å. Furthermore, two chelate carboxylate groups (Y—O distances in the range of 2.3847 (14)–2.4317 (14) Å), one N atom of 4,4'-bipy (Y—N distance 2.5357 (17) Å), one water molecule (Y—O distance 2.3884 (16) Å) complete the eight-coordinated configuration of Y atom. In addition, there are two lattice included non-coordinated 4,4'-bipy molecules in the crystal structure. There are extensive hydrogen-bonding interactions involving the Hca anions, 4,4'-bipy and coordinated water molecules (Table 1). Among these interactions, the distances between the carboxylate O atoms and hydroxyl oxygen are shorter than those for the others implying that these are `stronger' hydrogen bonds. These hydrogen bonds play a vital role in the construction of the extended three-dimensional supramolecular network (Fig. 2).

### Experimental

A mixture of Y(NO_3_)_3_.6H_2_O (0.1915 g, 0.5 mmol), 2-Hydroxycinnamic acid (0.2462 g, 1.5 mmol) and 4,4'-bipy (0.2343 g, 1.5 mmol) was dissolved in a 16 ml EtOH/H_2_O (*v*/*v*, 1:15 ml),and then sealed in a 25 ml Teflon-lined stainless steel reactor with a Telflon liner and heated at 433 K for 3 d. On completion of the reaction, the reactor was cooled slowly to room temperature and the mixture was filtered, giving colourless blocky single crystals suitable for X-ray analysis in 43% yield.

### Refinement

The Carbon-bound H atoms were positioned geometrically and included in the refinement using a riding model [C—H 0.93 Å *U*_iso_(H) = 1.2*U*_eq_(C)]. The water and hydroxyl H atoms were located from different maps, and their positions were refined isotropically, with O—H distances fixed by O_water_—H = 0.85 (2) Å, O_hydroxyl_—H = 0.96 (2) Å and H—H = 1.35 (2) Å, their displacement parameters were set to 1.5*U*_eq_(O_water_) and 1.2*U*_eq_(O_hydroxyl_).

### Crystal data

**Table:**

[Y_2_(C_9_H_7_O_3_)_6_(C_10_H_8_N_2_)_2_(H_2_O)_2_]·2C_10_H_8_N_2_	*Z* = 1
*M**_r_* = 1817.46	*F*(000) = 936
Triclinic, *P*1	*D*_x_ = 1.417 Mg m^−^^3^
Hall symbol: -P 1	Mo *K*α radiation, λ = 0.71073 Å
*a* = 11.8464 (7) Å	Cell parameters from 7095 reflections
*b* = 13.5272 (8) Å	θ = 1.5–27.7°
*c* = 13.7350 (8) Å	µ = 1.43 mm^−^^1^
α = 77.561 (3)°	*T* = 296 K
β = 88.850 (3)°	Block, colourless
γ = 82.283 (3)°	0.18 × 0.16 × 0.13 mm
*V* = 2129.8 (2) Å^3^	

### Data collection

**Table:**

Bruker APEXII area-detector diffractometer	9803 independent reflections
Radiation source: fine-focus sealed tube	7446 reflections with *I* > 2σ(*I*)
graphite	*R*_int_ = 0.041
φ and ω scans	θ_max_ = 27.7°, θ_min_ = 1.5°
Absorption correction: empirical (using intensity measurements) (*SADABS*; Sheldrick, 1996)	*h* = −15→15
*T*_min_ = 0.77, *T*_max_ = 0.83	*k* = −17→17
32270 measured reflections	*l* = −17→17

### Refinement

**Table:**

Refinement on *F*^2^	Primary atom site location: structure-invariant direct methods
Least-squares matrix: full	Secondary atom site location: difference Fourier map
*R*[*F*^2^ > 2σ(*F*^2^)] = 0.039	Hydrogen site location: inferred from neighbouring sites
*wR*(*F*^2^) = 0.087	H atoms treated by a mixture of independent and constrained refinement
*S* = 1.02	*w* = 1/[σ^2^(*F*_o_^2^) + (0.0384*P*)^2^ + 0.3503*P*] where *P* = (*F*_o_^2^ + 2*F*_c_^2^)/3
9803 reflections	(Δ/σ)_max_ = 0.001
574 parameters	Δρ_max_ = 0.24 e Å^−^^3^
6 restraints	Δρ_min_ = −0.43 e Å^−^^3^

### Special details

**Table:**

Geometry. All e.s.d.'s (except the e.s.d. in the dihedral angle between two l.s. planes) are estimated using the full covariance matrix. The cell e.s.d.'s are taken into account individually in the estimation of e.s.d.'s in distances, angles and torsion angles; correlations between e.s.d.'s in cell parameters are only used when they are defined by crystal symmetry. An approximate (isotropic) treatment of cell e.s.d.'s is used for estimating e.s.d.'s involving l.s. planes.
Refinement. Refinement of *F*^2^ against ALL reflections. The weighted *R*-factor *wR* and goodness of fit *S* are based on *F*^2^, conventional *R*-factors *R* are based on *F*, with *F* set to zero for negative *F*^2^. The threshold expression of *F*^2^ > σ(*F*^2^) is used only for calculating *R*-factors(gt) *etc*. and is not relevant to the choice of reflections for refinement. *R*-factors based on *F*^2^ are statistically about twice as large as those based on *F*, and *R*- factors based on ALL data will be even larger.

### Fractional atomic coordinates and isotropic or equivalent isotropic displacement parameters (Å^2^)

**Table:**

	*x*	*y*	*z*	*U*_iso_*/*U*_eq_	
Y1	0.855694 (16)	0.115806 (15)	0.037114 (15)	0.02694 (7)	
C1	0.85488 (17)	−0.09980 (17)	−0.00980 (16)	0.0313 (5)	
C2	0.81774 (18)	−0.20075 (17)	0.00737 (18)	0.0372 (5)	
H2A	0.8692	−0.2564	−0.0014	0.045*	
C3	0.71312 (18)	−0.21460 (17)	0.03498 (18)	0.0382 (5)	
H3A	0.6655	−0.1558	0.0412	0.046*	
C4	0.66089 (19)	−0.30778 (17)	0.05719 (18)	0.0394 (6)	
C5	0.7169 (2)	−0.4020 (2)	0.0459 (3)	0.0669 (9)	
H5A	0.7916	−0.4067	0.0234	0.080*	
C6	0.6635 (3)	−0.4879 (2)	0.0674 (3)	0.0843 (11)	
H6A	0.7019	−0.5504	0.0598	0.101*	
C7	0.5530 (3)	−0.4810 (2)	0.1002 (3)	0.0707 (9)	
H7A	0.5169	−0.5393	0.1146	0.085*	
C8	0.4956 (2)	−0.3901 (2)	0.1121 (2)	0.0541 (7)	
H8A	0.4206	−0.3865	0.1339	0.065*	
C9	0.54909 (19)	−0.30313 (18)	0.09145 (18)	0.0392 (5)	
C10	0.79402 (18)	0.16610 (15)	−0.16351 (16)	0.0302 (5)	
C11	0.75080 (18)	0.16500 (17)	−0.26180 (16)	0.0348 (5)	
H11A	0.6725	0.1772	−0.2731	0.042*	
C12	0.81864 (19)	0.14727 (17)	−0.33571 (16)	0.0365 (5)	
H12A	0.8962	0.1457	−0.3250	0.044*	
C13	0.78627 (19)	0.12985 (17)	−0.43196 (16)	0.0345 (5)	
C14	0.6792 (2)	0.10547 (19)	−0.44966 (18)	0.0432 (6)	
H14A	0.6238	0.1060	−0.4008	0.052*	
C15	0.6535 (2)	0.0805 (2)	−0.53829 (19)	0.0517 (7)	
H15A	0.5814	0.0647	−0.5492	0.062*	
C16	0.7360 (2)	0.0794 (2)	−0.61052 (19)	0.0533 (7)	
H16A	0.7196	0.0620	−0.6701	0.064*	
C17	0.8413 (2)	0.1036 (2)	−0.59547 (18)	0.0503 (7)	
H17A	0.8959	0.1029	−0.6450	0.060*	
C18	0.8679 (2)	0.12931 (18)	−0.50698 (18)	0.0407 (6)	
C19	0.90218 (18)	0.28475 (16)	0.10206 (17)	0.0323 (5)	
C20	0.95262 (19)	0.36770 (16)	0.13052 (17)	0.0370 (5)	
H20A	0.9658	0.4239	0.0814	0.044*	
C21	0.98010 (18)	0.36486 (16)	0.22470 (17)	0.0355 (5)	
H21A	0.9611	0.3086	0.2709	0.043*	
C22	1.03534 (19)	0.43649 (17)	0.26637 (17)	0.0365 (5)	
C23	1.0520 (2)	0.41712 (19)	0.36885 (18)	0.0463 (6)	
H23A	1.0255	0.3602	0.4084	0.056*	
C24	1.1064 (3)	0.4793 (2)	0.4138 (2)	0.0598 (8)	
H24A	1.1167	0.4642	0.4826	0.072*	
C25	1.1453 (3)	0.5639 (2)	0.3562 (2)	0.0640 (8)	
H25A	1.1821	0.6064	0.3860	0.077*	
C26	1.1301 (2)	0.58573 (19)	0.2551 (2)	0.0574 (8)	
H26A	1.1567	0.6432	0.2167	0.069*	
C27	1.0756 (2)	0.52335 (18)	0.20906 (18)	0.0442 (6)	
C28	0.5203 (2)	0.21910 (19)	0.2066 (2)	0.0488 (7)	
H28A	0.4974	0.2760	0.2335	0.059*	
C29	0.6227 (2)	0.21047 (19)	0.1584 (2)	0.0492 (7)	
H29A	0.6671	0.2630	0.1532	0.059*	
C30	0.5944 (2)	0.0591 (2)	0.12545 (19)	0.0476 (7)	
H30A	0.6187	0.0036	0.0970	0.057*	
C31	0.4906 (2)	0.06210 (19)	0.17222 (19)	0.0477 (6)	
H31A	0.4470	0.0094	0.1749	0.057*	
C32	0.45146 (18)	0.14325 (17)	0.21511 (16)	0.0334 (5)	
C33	0.34283 (18)	0.14721 (17)	0.27048 (16)	0.0348 (5)	
C34	0.25087 (19)	0.10641 (18)	0.24193 (18)	0.0401 (6)	
H34A	0.2563	0.0765	0.1868	0.048*	
C35	0.1513 (2)	0.11016 (19)	0.29536 (18)	0.0439 (6)	
H35A	0.0899	0.0839	0.2739	0.053*	
C36	0.2274 (2)	0.1885 (2)	0.40364 (19)	0.0517 (7)	
H36A	0.2200	0.2159	0.4604	0.062*	
C37	0.3294 (2)	0.1908 (2)	0.35354 (19)	0.0474 (6)	
H37A	0.3878	0.2209	0.3748	0.057*	
C38	0.3050 (3)	0.5444 (2)	0.6251 (2)	0.0644 (8)	
H38A	0.2258	0.5511	0.6259	0.077*	
C39	0.3596 (3)	0.6296 (2)	0.6092 (3)	0.0781 (10)	
H39A	0.3146	0.6929	0.5997	0.094*	
C40	0.5311 (3)	0.5367 (2)	0.6197 (2)	0.0680 (8)	
H40A	0.6101	0.5325	0.6180	0.082*	
C41	0.4845 (2)	0.4467 (2)	0.6360 (2)	0.0598 (7)	
H41A	0.5317	0.3846	0.6443	0.072*	
C42	0.3678 (2)	0.44854 (19)	0.64000 (19)	0.0473 (6)	
C43	0.3136 (2)	0.35367 (19)	0.66164 (19)	0.0469 (6)	
C44	0.3667 (3)	0.2638 (2)	0.6406 (2)	0.0621 (8)	
H44A	0.4367	0.2621	0.6086	0.075*	
C45	0.3156 (3)	0.1764 (2)	0.6670 (2)	0.0691 (9)	
H45A	0.3538	0.1168	0.6524	0.083*	
C46	0.1643 (3)	0.2586 (2)	0.7290 (2)	0.0654 (8)	
H46A	0.0929	0.2585	0.7584	0.078*	
C47	0.2090 (3)	0.3497 (2)	0.7062 (2)	0.0625 (8)	
H47A	0.1685	0.4084	0.7210	0.075*	
N1	0.66204 (15)	0.13120 (14)	0.11866 (13)	0.0344 (4)	
N2	0.13905 (16)	0.14964 (16)	0.37618 (15)	0.0449 (5)	
N3	0.4718 (2)	0.6286 (2)	0.6064 (2)	0.0741 (8)	
N4	0.2160 (2)	0.17128 (18)	0.71180 (18)	0.0598 (6)	
O1	0.78538 (13)	−0.02351 (12)	0.00007 (12)	0.0421 (4)	
O1W	0.90535 (14)	−0.01639 (11)	0.18130 (12)	0.0392 (4)	
H1WA	0.8654 (17)	−0.0576 (17)	0.2143 (18)	0.059*	
H1WB	0.9676 (14)	−0.0511 (18)	0.1776 (19)	0.059*	
O2	0.95636 (12)	−0.09016 (12)	−0.03507 (12)	0.0438 (4)	
O3	0.49790 (14)	−0.21136 (14)	0.10525 (15)	0.0548 (5)	
H3	0.4223 (15)	−0.213 (2)	0.107 (2)	0.066*	
O4	0.72411 (12)	0.18905 (11)	−0.09735 (11)	0.0347 (4)	
O5	0.89816 (12)	0.14171 (11)	−0.13982 (11)	0.0341 (3)	
O6	0.97039 (16)	0.15478 (17)	−0.48826 (14)	0.0640 (6)	
H6	1.019 (2)	0.150 (2)	−0.5393 (17)	0.077*	
O7	0.87559 (14)	0.29257 (11)	0.01038 (11)	0.0396 (4)	
O8	0.88615 (13)	0.20519 (11)	0.16396 (11)	0.0394 (4)	
O9	1.05892 (19)	0.54442 (14)	0.10954 (13)	0.0667 (6)	
H9	1.090 (2)	0.5997 (17)	0.077 (2)	0.080*	

### Atomic displacement parameters (Å^2^)

**Table:**

	*U*^11^	*U*^22^	*U*^33^	*U*^12^	*U*^13^	*U*^23^
Y1	0.02205 (11)	0.02963 (11)	0.03082 (12)	−0.00781 (8)	0.00578 (8)	−0.00801 (8)
C1	0.0242 (11)	0.0434 (13)	0.0299 (12)	−0.0105 (10)	0.0032 (9)	−0.0124 (10)
C2	0.0252 (11)	0.0333 (12)	0.0560 (16)	−0.0043 (9)	0.0063 (10)	−0.0159 (11)
C3	0.0293 (12)	0.0339 (12)	0.0535 (15)	−0.0057 (10)	0.0042 (11)	−0.0131 (11)
C4	0.0280 (12)	0.0346 (13)	0.0557 (16)	−0.0076 (10)	0.0033 (11)	−0.0081 (11)
C5	0.0367 (15)	0.0421 (16)	0.125 (3)	−0.0090 (12)	0.0115 (16)	−0.0235 (17)
C6	0.054 (2)	0.0366 (17)	0.162 (4)	−0.0062 (14)	0.004 (2)	−0.0219 (19)
C7	0.058 (2)	0.0433 (17)	0.108 (3)	−0.0230 (15)	0.0014 (18)	−0.0018 (16)
C8	0.0366 (14)	0.0595 (18)	0.0646 (19)	−0.0209 (13)	0.0047 (13)	−0.0016 (14)
C9	0.0295 (12)	0.0410 (14)	0.0467 (15)	−0.0104 (10)	0.0008 (10)	−0.0048 (11)
C10	0.0286 (12)	0.0284 (11)	0.0343 (13)	−0.0062 (9)	0.0070 (9)	−0.0077 (9)
C11	0.0279 (12)	0.0449 (14)	0.0331 (13)	−0.0054 (10)	0.0027 (10)	−0.0112 (10)
C12	0.0349 (13)	0.0410 (13)	0.0344 (13)	−0.0079 (10)	0.0062 (10)	−0.0083 (10)
C13	0.0370 (13)	0.0373 (13)	0.0296 (12)	−0.0047 (10)	0.0042 (10)	−0.0086 (10)
C14	0.0434 (14)	0.0523 (15)	0.0351 (14)	−0.0087 (12)	0.0068 (11)	−0.0111 (11)
C15	0.0524 (16)	0.0611 (17)	0.0453 (16)	−0.0169 (13)	−0.0020 (13)	−0.0133 (13)
C16	0.073 (2)	0.0581 (17)	0.0314 (14)	−0.0142 (14)	−0.0013 (13)	−0.0129 (12)
C17	0.0599 (18)	0.0602 (17)	0.0322 (14)	−0.0095 (14)	0.0138 (12)	−0.0133 (12)
C18	0.0403 (14)	0.0466 (14)	0.0356 (14)	−0.0077 (11)	0.0100 (11)	−0.0098 (11)
C19	0.0302 (12)	0.0314 (12)	0.0362 (14)	−0.0056 (9)	0.0037 (10)	−0.0087 (10)
C20	0.0445 (14)	0.0308 (12)	0.0374 (14)	−0.0125 (10)	−0.0005 (11)	−0.0061 (10)
C21	0.0388 (13)	0.0304 (12)	0.0365 (14)	−0.0060 (10)	0.0025 (10)	−0.0050 (10)
C22	0.0414 (13)	0.0329 (12)	0.0358 (13)	−0.0043 (10)	−0.0035 (10)	−0.0092 (10)
C23	0.0602 (17)	0.0436 (15)	0.0351 (14)	−0.0094 (12)	−0.0001 (12)	−0.0067 (11)
C24	0.081 (2)	0.0647 (19)	0.0384 (16)	−0.0166 (16)	−0.0114 (14)	−0.0152 (13)
C25	0.083 (2)	0.0558 (18)	0.063 (2)	−0.0227 (16)	−0.0197 (17)	−0.0230 (15)
C26	0.080 (2)	0.0408 (15)	0.0557 (18)	−0.0258 (14)	−0.0111 (15)	−0.0071 (13)
C27	0.0567 (16)	0.0387 (14)	0.0384 (15)	−0.0126 (12)	−0.0078 (12)	−0.0062 (11)
C28	0.0415 (14)	0.0401 (14)	0.0697 (18)	−0.0071 (11)	0.0208 (13)	−0.0236 (13)
C29	0.0395 (14)	0.0410 (14)	0.0712 (19)	−0.0131 (11)	0.0196 (13)	−0.0184 (13)
C30	0.0404 (14)	0.0539 (16)	0.0591 (17)	−0.0148 (12)	0.0224 (12)	−0.0323 (13)
C31	0.0408 (14)	0.0523 (16)	0.0610 (17)	−0.0211 (12)	0.0231 (12)	−0.0289 (13)
C32	0.0278 (11)	0.0400 (13)	0.0312 (12)	−0.0011 (9)	0.0054 (9)	−0.0073 (10)
C33	0.0298 (12)	0.0376 (13)	0.0348 (13)	−0.0029 (10)	0.0089 (10)	−0.0048 (10)
C34	0.0339 (13)	0.0492 (15)	0.0380 (14)	−0.0066 (11)	0.0075 (10)	−0.0114 (11)
C35	0.0329 (13)	0.0528 (15)	0.0468 (16)	−0.0080 (11)	0.0086 (11)	−0.0117 (12)
C36	0.0432 (15)	0.0711 (19)	0.0448 (16)	−0.0010 (13)	0.0129 (12)	−0.0262 (14)
C37	0.0350 (14)	0.0633 (17)	0.0499 (16)	−0.0068 (12)	0.0072 (12)	−0.0259 (13)
C38	0.0583 (19)	0.0477 (17)	0.087 (2)	−0.0085 (14)	−0.0032 (16)	−0.0132 (15)
C39	0.083 (3)	0.0433 (18)	0.107 (3)	−0.0095 (17)	−0.003 (2)	−0.0139 (17)
C40	0.0600 (19)	0.066 (2)	0.084 (2)	−0.0198 (16)	0.0074 (16)	−0.0220 (17)
C41	0.0568 (19)	0.0515 (17)	0.072 (2)	−0.0071 (14)	0.0044 (15)	−0.0145 (14)
C42	0.0543 (17)	0.0445 (15)	0.0437 (15)	−0.0103 (12)	−0.0002 (12)	−0.0086 (11)
C43	0.0530 (16)	0.0418 (15)	0.0435 (15)	−0.0056 (12)	−0.0042 (12)	−0.0043 (11)
C44	0.0626 (19)	0.0506 (17)	0.075 (2)	−0.0148 (14)	0.0149 (16)	−0.0138 (15)
C45	0.082 (2)	0.0460 (17)	0.082 (2)	−0.0144 (16)	0.0139 (19)	−0.0163 (15)
C46	0.0580 (19)	0.061 (2)	0.073 (2)	−0.0144 (15)	0.0144 (16)	−0.0024 (16)
C47	0.0607 (19)	0.0456 (16)	0.077 (2)	−0.0049 (14)	0.0115 (16)	−0.0073 (14)
N1	0.0287 (10)	0.0413 (11)	0.0335 (11)	−0.0077 (8)	0.0077 (8)	−0.0078 (8)
N2	0.0329 (11)	0.0554 (13)	0.0436 (13)	−0.0020 (9)	0.0134 (9)	−0.0082 (10)
N3	0.078 (2)	0.0586 (17)	0.092 (2)	−0.0252 (15)	0.0038 (16)	−0.0210 (14)
N4	0.0672 (16)	0.0496 (15)	0.0607 (16)	−0.0169 (12)	0.0058 (13)	−0.0026 (11)
O1	0.0378 (9)	0.0371 (9)	0.0579 (11)	−0.0117 (7)	0.0155 (8)	−0.0214 (8)
O1W	0.0356 (9)	0.0345 (9)	0.0434 (10)	−0.0032 (7)	0.0126 (8)	−0.0016 (7)
O2	0.0232 (8)	0.0552 (11)	0.0524 (11)	−0.0140 (7)	0.0030 (7)	−0.0052 (8)
O3	0.0249 (9)	0.0573 (12)	0.0887 (14)	−0.0105 (8)	0.0123 (9)	−0.0277 (10)
O4	0.0255 (8)	0.0467 (9)	0.0341 (9)	−0.0023 (7)	0.0061 (6)	−0.0151 (7)
O5	0.0244 (8)	0.0434 (9)	0.0348 (9)	−0.0039 (7)	0.0068 (6)	−0.0098 (7)
O6	0.0425 (11)	0.1103 (17)	0.0502 (12)	−0.0252 (11)	0.0212 (9)	−0.0342 (11)
O7	0.0542 (10)	0.0331 (9)	0.0328 (9)	−0.0128 (7)	−0.0063 (8)	−0.0054 (7)
O8	0.0505 (10)	0.0374 (9)	0.0339 (9)	−0.0196 (8)	0.0055 (7)	−0.0076 (7)
O9	0.1122 (17)	0.0543 (12)	0.0388 (11)	−0.0483 (12)	−0.0121 (11)	0.0028 (9)

### Geometric parameters (Å, °)

**Table:**

Y1—O2^i^	2.2074 (15)	C23—H23A	0.9300
Y1—O1	2.3143 (15)	C24—C25	1.373 (4)
Y1—O7	2.3827 (14)	C24—H24A	0.9300
Y1—O8	2.3827 (15)	C25—C26	1.366 (4)
Y1—O1W	2.3884 (16)	C25—H25A	0.9300
Y1—O4	2.3948 (15)	C26—C27	1.387 (3)
Y1—O5	2.4317 (14)	C26—H26A	0.9300
Y1—N1	2.5357 (17)	C27—O9	1.348 (3)
Y1—C19	2.749 (2)	C28—C29	1.374 (3)
Y1—C10	2.780 (2)	C28—C32	1.379 (3)
C1—O2	1.260 (2)	C28—H28A	0.9300
C1—O1	1.260 (3)	C29—N1	1.332 (3)
C1—C2	1.460 (3)	C29—H29A	0.9300
C2—C3	1.315 (3)	C30—N1	1.330 (3)
C2—H2A	0.9300	C30—C31	1.376 (3)
C3—C4	1.449 (3)	C30—H30A	0.9300
C3—H3A	0.9300	C31—C32	1.378 (3)
C4—C5	1.394 (3)	C31—H31A	0.9300
C4—C9	1.395 (3)	C32—C33	1.482 (3)
C5—C6	1.370 (4)	C33—C34	1.385 (3)
C5—H5A	0.9300	C33—C37	1.390 (3)
C6—C7	1.374 (4)	C34—C35	1.378 (3)
C6—H6A	0.9300	C34—H34A	0.9300
C7—C8	1.364 (4)	C35—N2	1.329 (3)
C7—H7A	0.9300	C35—H35A	0.9300
C8—C9	1.384 (3)	C36—N2	1.328 (3)
C8—H8A	0.9300	C36—C37	1.379 (3)
C9—O3	1.356 (3)	C36—H36A	0.9300
C10—O5	1.264 (2)	C37—H37A	0.9300
C10—O4	1.277 (2)	C38—C39	1.372 (4)
C10—C11	1.457 (3)	C38—C42	1.382 (4)
C11—C12	1.325 (3)	C38—H38A	0.9300
C11—H11A	0.9300	C39—N3	1.327 (4)
C12—C13	1.459 (3)	C39—H39A	0.9300
C12—H12A	0.9300	C40—N3	1.320 (4)
C13—C14	1.391 (3)	C40—C41	1.377 (4)
C13—C18	1.399 (3)	C40—H40A	0.9300
C14—C15	1.381 (3)	C41—C42	1.380 (4)
C14—H14A	0.9300	C41—H41A	0.9300
C15—C16	1.380 (4)	C42—C43	1.482 (3)
C15—H15A	0.9300	C43—C47	1.374 (4)
C16—C17	1.362 (4)	C43—C44	1.378 (4)
C16—H16A	0.9300	C44—C45	1.376 (4)
C17—C18	1.387 (3)	C44—H44A	0.9300
C17—H17A	0.9300	C45—N4	1.323 (4)
C18—O6	1.350 (3)	C45—H45A	0.9300
C19—O8	1.254 (2)	C46—N4	1.321 (4)
C19—O7	1.283 (3)	C46—C47	1.379 (4)
C19—C20	1.465 (3)	C46—H46A	0.9300
C20—C21	1.331 (3)	C47—H47A	0.9300
C20—H20A	0.9300	O1W—H1WA	0.833 (16)
C21—C22	1.456 (3)	O1W—H1WB	0.826 (16)
C21—H21A	0.9300	O2—Y1^i^	2.2074 (15)
C22—C23	1.388 (3)	O3—H3	0.898 (17)
C22—C27	1.402 (3)	O6—H6	0.909 (17)
C23—C24	1.374 (3)	O9—H9	0.904 (17)
			
O2^i^—Y1—O1	109.25 (6)	O8—C19—Y1	59.95 (11)
O2^i^—Y1—O7	85.57 (6)	O7—C19—Y1	60.01 (10)
O1—Y1—O7	153.90 (6)	C20—C19—Y1	167.40 (15)
O2^i^—Y1—O8	83.23 (6)	C21—C20—C19	121.9 (2)
O1—Y1—O8	145.87 (5)	C21—C20—H20A	119.1
O7—Y1—O8	54.49 (5)	C19—C20—H20A	119.1
O2^i^—Y1—O1W	76.14 (6)	C20—C21—C22	129.8 (2)
O1—Y1—O1W	76.64 (6)	C20—C21—H21A	115.1
O7—Y1—O1W	128.80 (5)	C22—C21—H21A	115.1
O8—Y1—O1W	75.87 (5)	C23—C22—C27	117.4 (2)
O2^i^—Y1—O4	129.23 (5)	C23—C22—C21	118.6 (2)
O1—Y1—O4	75.98 (6)	C27—C22—C21	124.0 (2)
O7—Y1—O4	78.03 (5)	C24—C23—C22	122.2 (2)
O8—Y1—O4	121.30 (5)	C24—C23—H23A	118.9
O1W—Y1—O4	147.86 (5)	C22—C23—H23A	118.9
O2^i^—Y1—O5	76.88 (5)	C25—C24—C23	119.4 (3)
O1—Y1—O5	80.03 (5)	C25—C24—H24A	120.3
O7—Y1—O5	82.79 (5)	C23—C24—H24A	120.3
O8—Y1—O5	134.09 (5)	C26—C25—C24	120.1 (2)
O1W—Y1—O5	135.74 (5)	C26—C25—H25A	119.9
O4—Y1—O5	53.74 (5)	C24—C25—H25A	119.9
O2^i^—Y1—N1	155.07 (6)	C25—C26—C27	120.9 (2)
O1—Y1—N1	79.86 (5)	C25—C26—H26A	119.5
O7—Y1—N1	95.71 (6)	C27—C26—H26A	119.5
O8—Y1—N1	77.47 (6)	O9—C27—C26	121.6 (2)
O1W—Y1—N1	83.90 (6)	O9—C27—C22	118.4 (2)
O4—Y1—N1	75.01 (5)	C26—C27—C22	120.0 (2)
O5—Y1—N1	128.02 (5)	C29—C28—C32	119.9 (2)
O2^i^—Y1—C19	80.28 (6)	C29—C28—H28A	120.1
O1—Y1—C19	169.10 (6)	C32—C28—H28A	120.1
O7—Y1—C19	27.79 (6)	N1—C29—C28	123.8 (2)
O8—Y1—C19	27.09 (6)	N1—C29—H29A	118.1
O1W—Y1—C19	101.28 (6)	C28—C29—H29A	118.1
O4—Y1—C19	102.40 (6)	N1—C30—C31	123.7 (2)
O5—Y1—C19	107.92 (6)	N1—C30—H30A	118.1
N1—Y1—C19	89.29 (6)	C31—C30—H30A	118.1
O2^i^—Y1—C10	103.85 (6)	C30—C31—C32	119.9 (2)
O1—Y1—C10	72.50 (6)	C30—C31—H31A	120.0
O7—Y1—C10	83.30 (6)	C32—C31—H31A	120.0
O8—Y1—C10	136.88 (6)	C31—C32—C28	116.6 (2)
O1W—Y1—C10	147.24 (6)	C31—C32—C33	121.8 (2)
O4—Y1—C10	27.29 (5)	C28—C32—C33	121.6 (2)
O5—Y1—C10	27.02 (5)	C34—C33—C37	117.3 (2)
N1—Y1—C10	101.02 (6)	C34—C33—C32	121.1 (2)
C19—Y1—C10	111.08 (6)	C37—C33—C32	121.6 (2)
O2—C1—O1	120.8 (2)	C35—C34—C33	119.9 (2)
O2—C1—C2	119.2 (2)	C35—C34—H34A	120.0
O1—C1—C2	119.90 (19)	C33—C34—H34A	120.0
C3—C2—C1	121.3 (2)	N2—C35—C34	122.9 (2)
C3—C2—H2A	119.3	N2—C35—H35A	118.5
C1—C2—H2A	119.3	C34—C35—H35A	118.5
C2—C3—C4	129.7 (2)	N2—C36—C37	124.3 (2)
C2—C3—H3A	115.2	N2—C36—H36A	117.9
C4—C3—H3A	115.2	C37—C36—H36A	117.9
C5—C4—C9	118.2 (2)	C36—C37—C33	118.5 (2)
C5—C4—C3	123.2 (2)	C36—C37—H37A	120.8
C9—C4—C3	118.5 (2)	C33—C37—H37A	120.8
C6—C5—C4	121.0 (3)	C39—C38—C42	119.9 (3)
C6—C5—H5A	119.5	C39—C38—H38A	120.1
C4—C5—H5A	119.5	C42—C38—H38A	120.1
C5—C6—C7	119.5 (3)	N3—C39—C38	124.9 (3)
C5—C6—H6A	120.2	N3—C39—H39A	117.6
C7—C6—H6A	120.2	C38—C39—H39A	117.6
C8—C7—C6	121.1 (3)	N3—C40—C41	124.8 (3)
C8—C7—H7A	119.5	N3—C40—H40A	117.6
C6—C7—H7A	119.5	C41—C40—H40A	117.6
C7—C8—C9	119.8 (2)	C40—C41—C42	119.9 (3)
C7—C8—H8A	120.1	C40—C41—H41A	120.0
C9—C8—H8A	120.1	C42—C41—H41A	120.0
O3—C9—C8	122.6 (2)	C41—C42—C38	115.7 (2)
O3—C9—C4	117.1 (2)	C41—C42—C43	122.0 (2)
C8—C9—C4	120.3 (2)	C38—C42—C43	122.3 (2)
O5—C10—O4	118.35 (19)	C47—C43—C44	116.2 (2)
O5—C10—C11	122.43 (19)	C47—C43—C42	121.9 (2)
O4—C10—C11	119.18 (19)	C44—C43—C42	121.9 (2)
O5—C10—Y1	60.94 (11)	C45—C44—C43	119.6 (3)
O4—C10—Y1	59.30 (11)	C45—C44—H44A	120.2
C11—C10—Y1	164.18 (15)	C43—C44—H44A	120.2
C12—C11—C10	122.6 (2)	N4—C45—C44	124.7 (3)
C12—C11—H11A	118.7	N4—C45—H45A	117.7
C10—C11—H11A	118.7	C44—C45—H45A	117.7
C11—C12—C13	127.8 (2)	N4—C46—C47	124.4 (3)
C11—C12—H12A	116.1	N4—C46—H46A	117.8
C13—C12—H12A	116.1	C47—C46—H46A	117.8
C14—C13—C18	118.2 (2)	C43—C47—C46	119.8 (3)
C14—C13—C12	122.3 (2)	C43—C47—H47A	120.1
C18—C13—C12	119.3 (2)	C46—C47—H47A	120.1
C15—C14—C13	121.4 (2)	C30—N1—C29	116.08 (19)
C15—C14—H14A	119.3	C30—N1—Y1	121.52 (15)
C13—C14—H14A	119.3	C29—N1—Y1	122.39 (14)
C16—C15—C14	119.2 (2)	C36—N2—C35	117.1 (2)
C16—C15—H15A	120.4	C40—N3—C39	114.8 (3)
C14—C15—H15A	120.4	C46—N4—C45	115.2 (2)
C17—C16—C15	120.7 (2)	C1—O1—Y1	118.61 (13)
C17—C16—H16A	119.7	Y1—O1W—H1WA	129.3 (18)
C15—C16—H16A	119.7	Y1—O1W—H1WB	114.1 (18)
C16—C17—C18	120.6 (2)	H1WA—O1W—H1WB	104 (2)
C16—C17—H17A	119.7	C1—O2—Y1^i^	161.16 (15)
C18—C17—H17A	119.7	C9—O3—H3	108.1 (17)
O6—C18—C17	123.2 (2)	C10—O4—Y1	93.42 (12)
O6—C18—C13	116.9 (2)	C10—O5—Y1	92.04 (12)
C17—C18—C13	119.9 (2)	C18—O6—H6	111.6 (18)
O8—C19—O7	118.66 (19)	C19—O7—Y1	92.20 (12)
O8—C19—C20	122.2 (2)	C19—O8—Y1	92.96 (13)
O7—C19—C20	119.08 (19)	C27—O9—H9	113.7 (19)
			
O2—C1—C2—C3	179.2 (2)	C28—C32—C33—C34	147.9 (2)
O1—C1—C2—C3	−1.3 (3)	C31—C32—C33—C37	145.1 (3)
C1—C2—C3—C4	−178.8 (2)	C28—C32—C33—C37	−32.8 (3)
C2—C3—C4—C5	−4.2 (4)	C37—C33—C34—C35	0.0 (3)
C2—C3—C4—C9	175.9 (2)	C32—C33—C34—C35	179.3 (2)
C9—C4—C5—C6	0.3 (5)	C33—C34—C35—N2	−1.6 (4)
C3—C4—C5—C6	−179.7 (3)	N2—C36—C37—C33	−2.0 (4)
C4—C5—C6—C7	0.3 (6)	C34—C33—C37—C36	1.7 (4)
C5—C6—C7—C8	−0.2 (6)	C32—C33—C37—C36	−177.6 (2)
C6—C7—C8—C9	−0.5 (5)	C42—C38—C39—N3	0.2 (5)
C7—C8—C9—O3	−177.5 (3)	N3—C40—C41—C42	−0.5 (5)
C7—C8—C9—C4	1.0 (4)	C40—C41—C42—C38	1.1 (4)
C5—C4—C9—O3	177.7 (2)	C40—C41—C42—C43	−177.3 (3)
C3—C4—C9—O3	−2.4 (3)	C39—C38—C42—C41	−0.9 (4)
C5—C4—C9—C8	−0.9 (4)	C39—C38—C42—C43	177.4 (3)
C3—C4—C9—C8	179.0 (2)	C41—C42—C43—C47	152.0 (3)
O2^i^—Y1—C10—O5	3.52 (13)	C38—C42—C43—C47	−26.2 (4)
O1—Y1—C10—O5	−102.64 (12)	C41—C42—C43—C44	−26.2 (4)
O7—Y1—C10—O5	87.26 (12)	C38—C42—C43—C44	155.5 (3)
O8—Y1—C10—O5	98.43 (13)	C47—C43—C44—C45	−1.9 (4)
O1W—Y1—C10—O5	−82.29 (15)	C42—C43—C44—C45	176.5 (3)
O4—Y1—C10—O5	164.1 (2)	C43—C44—C45—N4	0.6 (5)
N1—Y1—C10—O5	−178.19 (11)	C44—C43—C47—C46	1.3 (4)
C19—Y1—C10—O5	88.28 (12)	C42—C43—C47—C46	−177.1 (3)
O2^i^—Y1—C10—O4	−160.56 (11)	N4—C46—C47—C43	0.7 (5)
O1—Y1—C10—O4	93.28 (12)	C31—C30—N1—C29	−1.5 (4)
O7—Y1—C10—O4	−76.82 (12)	C31—C30—N1—Y1	177.5 (2)
O8—Y1—C10—O4	−65.65 (14)	C28—C29—N1—C30	1.7 (4)
O1W—Y1—C10—O4	113.62 (13)	C28—C29—N1—Y1	−177.3 (2)
O5—Y1—C10—O4	−164.1 (2)	O2^i^—Y1—N1—C30	−106.9 (2)
N1—Y1—C10—O4	17.73 (13)	O1—Y1—N1—C30	7.31 (18)
C19—Y1—C10—O4	−75.81 (12)	O7—Y1—N1—C30	161.33 (18)
O2^i^—Y1—C10—C11	110.3 (6)	O8—Y1—N1—C30	−147.00 (19)
O1—Y1—C10—C11	4.2 (5)	O1W—Y1—N1—C30	−70.16 (19)
O7—Y1—C10—C11	−165.9 (6)	O4—Y1—N1—C30	85.37 (18)
O8—Y1—C10—C11	−154.7 (5)	O5—Y1—N1—C30	76.0 (2)
O1W—Y1—C10—C11	24.5 (6)	C19—Y1—N1—C30	−171.59 (19)
O4—Y1—C10—C11	−89.1 (6)	C10—Y1—N1—C30	77.07 (19)
O5—Y1—C10—C11	106.8 (6)	O2^i^—Y1—N1—C29	72.1 (2)
N1—Y1—C10—C11	−71.4 (6)	O1—Y1—N1—C29	−173.75 (19)
C19—Y1—C10—C11	−164.9 (5)	O7—Y1—N1—C29	−19.73 (19)
O5—C10—C11—C12	−6.2 (3)	O8—Y1—N1—C29	31.94 (18)
O4—C10—C11—C12	176.3 (2)	O1W—Y1—N1—C29	108.78 (19)
Y1—C10—C11—C12	−103.7 (5)	O4—Y1—N1—C29	−95.69 (19)
C10—C11—C12—C13	170.6 (2)	O5—Y1—N1—C29	−105.04 (19)
C11—C12—C13—C14	−15.9 (4)	C19—Y1—N1—C29	7.35 (19)
C11—C12—C13—C18	169.4 (2)	C10—Y1—N1—C29	−104.00 (19)
C18—C13—C14—C15	0.5 (4)	C37—C36—N2—C35	0.4 (4)
C12—C13—C14—C15	−174.3 (2)	C34—C35—N2—C36	1.4 (4)
C13—C14—C15—C16	0.3 (4)	C41—C40—N3—C39	−0.3 (5)
C14—C15—C16—C17	−0.7 (4)	C38—C39—N3—C40	0.4 (5)
C15—C16—C17—C18	0.3 (4)	C47—C46—N4—C45	−2.0 (5)
C16—C17—C18—O6	−179.4 (2)	C44—C45—N4—C46	1.3 (5)
C16—C17—C18—C13	0.4 (4)	O2—C1—O1—Y1	−25.4 (3)
C14—C13—C18—O6	179.0 (2)	C2—C1—O1—Y1	155.05 (16)
C12—C13—C18—O6	−6.1 (3)	O2^i^—Y1—O1—C1	5.03 (17)
C14—C13—C18—C17	−0.8 (4)	O7—Y1—O1—C1	126.82 (17)
C12—C13—C18—C17	174.1 (2)	O8—Y1—O1—C1	−101.99 (17)
O2^i^—Y1—C19—O8	−94.15 (13)	O1W—Y1—O1—C1	−64.87 (16)
O1—Y1—C19—O8	57.4 (4)	O4—Y1—O1—C1	132.13 (16)
O7—Y1—C19—O8	166.8 (2)	O5—Y1—O1—C1	77.24 (16)
O1W—Y1—C19—O8	−20.54 (13)	N1—Y1—O1—C1	−150.95 (16)
O4—Y1—C19—O8	137.58 (12)	C19—Y1—O1—C1	−145.1 (3)
O5—Y1—C19—O8	−166.85 (12)	C10—Y1—O1—C1	103.98 (16)
N1—Y1—C19—O8	63.10 (13)	O1—C1—O2—Y1^i^	131.2 (4)
C10—Y1—C19—O8	164.65 (12)	C2—C1—O2—Y1^i^	−49.3 (6)
O2^i^—Y1—C19—O7	99.04 (13)	O5—C10—O4—Y1	−15.81 (19)
O1—Y1—C19—O7	−109.5 (3)	C11—C10—O4—Y1	161.81 (17)
O8—Y1—C19—O7	−166.8 (2)	O2^i^—Y1—O4—C10	24.65 (14)
O1W—Y1—C19—O7	172.66 (12)	O1—Y1—O4—C10	−78.93 (12)
O4—Y1—C19—O7	−29.23 (13)	O7—Y1—O4—C10	98.69 (12)
O5—Y1—C19—O7	26.35 (13)	O8—Y1—O4—C10	133.22 (11)
N1—Y1—C19—O7	−103.70 (12)	O1W—Y1—O4—C10	−111.25 (13)
C10—Y1—C19—O7	−2.16 (14)	O5—Y1—O4—C10	8.89 (11)
O2^i^—Y1—C19—C20	9.6 (7)	N1—Y1—O4—C10	−161.98 (13)
O1—Y1—C19—C20	161.1 (6)	C19—Y1—O4—C10	112.15 (12)
O7—Y1—C19—C20	−89.5 (7)	O4—C10—O5—Y1	15.54 (19)
O8—Y1—C19—C20	103.7 (8)	C11—C10—O5—Y1	−161.99 (18)
O1W—Y1—C19—C20	83.2 (7)	O2^i^—Y1—O5—C10	−176.49 (13)
O4—Y1—C19—C20	−118.7 (7)	O1—Y1—O5—C10	70.89 (12)
O5—Y1—C19—C20	−63.1 (7)	O7—Y1—O5—C10	−89.38 (12)
N1—Y1—C19—C20	166.8 (7)	O8—Y1—O5—C10	−109.71 (12)
C10—Y1—C19—C20	−91.6 (7)	O1W—Y1—O5—C10	129.79 (12)
O8—C19—C20—C21	−2.7 (3)	O4—Y1—O5—C10	−8.97 (11)
O7—C19—C20—C21	178.8 (2)	N1—Y1—O5—C10	2.25 (14)
Y1—C19—C20—C21	−98.9 (7)	C19—Y1—O5—C10	−101.42 (12)
C19—C20—C21—C22	176.6 (2)	O8—C19—O7—Y1	−13.0 (2)
C20—C21—C22—C23	178.3 (2)	C20—C19—O7—Y1	165.54 (18)
C20—C21—C22—C27	−3.2 (4)	O2^i^—Y1—O7—C19	−77.50 (13)
C27—C22—C23—C24	−0.5 (4)	O1—Y1—O7—C19	156.09 (13)
C21—C22—C23—C24	178.1 (2)	O8—Y1—O7—C19	7.34 (12)
C22—C23—C24—C25	0.3 (4)	O1W—Y1—O7—C19	−9.26 (15)
C23—C24—C25—C26	−0.1 (5)	O4—Y1—O7—C19	150.83 (13)
C24—C25—C26—C27	0.0 (5)	O5—Y1—O7—C19	−154.81 (13)
C25—C26—C27—O9	179.3 (3)	N1—Y1—O7—C19	77.50 (13)
C25—C26—C27—C22	−0.1 (4)	C10—Y1—O7—C19	177.97 (13)
C23—C22—C27—O9	−179.1 (2)	O7—C19—O8—Y1	13.0 (2)
C21—C22—C27—O9	2.4 (4)	C20—C19—O8—Y1	−165.49 (19)
C23—C22—C27—C26	0.3 (4)	O2^i^—Y1—O8—C19	81.87 (13)
C21—C22—C27—C26	−178.1 (2)	O1—Y1—O8—C19	−163.51 (12)
C32—C28—C29—N1	−0.6 (4)	O7—Y1—O8—C19	−7.51 (12)
N1—C30—C31—C32	0.2 (4)	O1W—Y1—O8—C19	159.22 (14)
C30—C31—C32—C28	1.0 (4)	O4—Y1—O8—C19	−50.45 (14)
C30—C31—C32—C33	−177.0 (2)	O5—Y1—O8—C19	17.54 (16)
C29—C28—C32—C31	−0.8 (4)	N1—Y1—O8—C19	−114.00 (14)
C29—C28—C32—C33	177.2 (2)	C10—Y1—O8—C19	−21.19 (16)
C31—C32—C33—C34	−34.2 (3)		

Symmetry codes: (i) −*x*+2, −*y*, −*z*.

### Hydrogen-bond geometry (Å, °)

**Table:**

*D*—H···*A*	*D*—H	H···*A*	*D*···*A*	*D*—H···*A*
O1W—H1WA···N4^ii^	0.83 (2)	2.00 (2)	2.830 (3)	172 (2)
O9—H9···O7^iii^	0.90 (2)	1.77 (2)	2.650 (2)	165 (3)
O1W—H1WB···O5^i^	0.83 (2)	2.00 (2)	2.814 (2)	166 (3)
O6—H6···N2^iv^	0.91 (2)	1.81 (2)	2.708 (3)	167 (3)
O3—H3···O4^v^	0.90 (2)	1.72 (2)	2.609 (2)	168 (3)

Symmetry codes: (ii) −*x*+1, −*y*, −*z*+1; (iii) −*x*+2, −*y*+1, −*z*; (i) −*x*+2, −*y*, −*z*; (iv) *x*+1, *y*, *z*−1; (v) −*x*+1, −*y*, −*z*.
